# Human mid-trimester amniotic fluid (stem) cells lack expression of the pluripotency marker OCT4A

**DOI:** 10.1038/s41598-019-44572-x

**Published:** 2019-05-31

**Authors:** Filipa Vlahova, Kate E. Hawkins, Anna Maria Ranzoni, Kwan-Leong Hau, Rachel Sagar, Paolo De Coppi, Anna L. David, James Adjaye, Pascale V. Guillot

**Affiliations:** 1 University College London, Institute for Women’s Health, Maternal and Fetal Medicine Department, London, UK; 2 University College London, Great Ormond Street Institute for Child Health, London, UK; 3 Department of Development and Regeneration, Katholieke Universiteit Leuven, Leuven, Belgium; 4 Specialist Neonatal and Paediatric Surgery, Great Ormond Street Hospital NHS Trust, London, UK; 5 NIHR University College London Hospitals Biomedical Research Centre, Maple House, 149 Tottenham Court Road, London, W1T 7DN UK; 6 Institute for Stem Cell Research and Regenerative Medicine, Medical Faculty, Heinrich Heine University, Düsseldorf, Moorenstr. 5, 40225 Düsseldorf, Germany

**Keywords:** Adult stem cells, Multipotent stem cells

## Abstract

Expression of OCT4A is one of the hallmarks of pluripotency, defined as a stem cell’s ability to differentiate into all the lineages of the three germ layers. Despite being defined as non-tumorigenic cells with high translational potential, human mid-trimester amniotic fluid stem cells (hAFSCs) are often described as sharing features with embryonic stem cells, including the expression of OCT4A, which could hinder their clinical potential. To clarify the OCT4A status of hAFSCs, we first undertook a systematic review of the literature. We then performed extensive gene and protein expression analyses to discover that neither frozen, nor fresh hAFSCs cultivated in multipotent stem cell culture conditions expressed OCT4A, and that the OCT4A positive results from the literature are likely to be attributed to the expression of pseudogenes or other OCT4 variants. To address this issue, we provide a robust protocol for the assessment of OCT4A in other stem cells.

## Introduction

**Figure 1:**
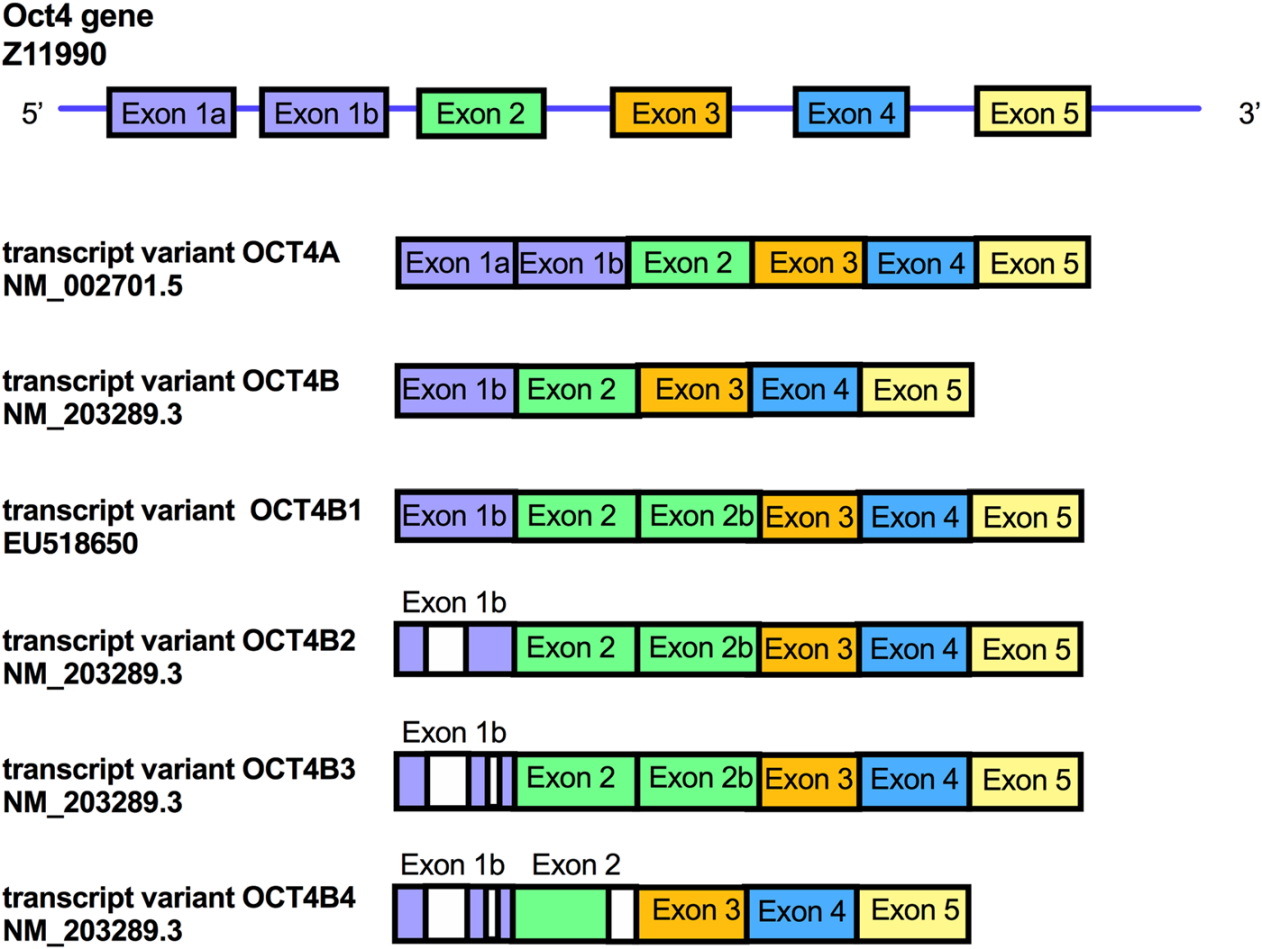
Schematic representation of the human OCT4 gene. List of transcript variants for OCT4. The OCT4A transcript variant is composed of exon 1a, and exons 2 to 5. The OCT4B transcript variant contains exon 1b, and exons 2, 3, 4 and 5. The OCT4B1 transcript variant contains exon 1b, exon 2, exon 2b, exon 3, 4 and 5. The OCT4B2 transcript variant is composed of exon 1b lacking a 630-nt fragment, 2, 2b, 3, 4 and 5. The OCT4B3 transcript variant is composed of exons 1b lacking a 207-nt fragment, exon 2, 2b, 3, 4 and 5. The OCTB4 transcript variant is composed of exon 1b, The OCT4B transcript variant contains exon 1b, exon 2 lacking a 28-nt fragment, exons 3, 4 and 5.

Pluripotency is defined by a number of stringent criteria, including the expression of octamer-binding transcription factor 4 (OCT4), NANOG and sex determining region Y-box 2 (SOX2), the ability to self-renew through symmetrical cell division, and the potential to form well-differentiated teratomas following injection into immuno-compromised mice^1^. Human OCT4 is encoded by the POU domain class 5 transcription factor 1 (POU5F1) gene, located on chromosome 6p21.3, which consists of 5 exons (Fig. 1)^2^. It is alternatively spliced to encode six transcripts variants, i.e. OCT4A^3,4^, OCT4B^3,4^, OCT4B1^5^, OCT4B2^5^, OCT4B3^6^ and OCT4B4^7^ (Fig. 1).

The OCT4A transcript variant is composed of exon 1a, and exons 2 to 5^3,4^. OCT4A (360 amino acids, 39 kDa^8^) is a transcription factor located in the nucleus of pluripotent stem cells that specifically binds to the conserved octamer motif ATTTGCAT on the regulatory regions of its downstream targets^9^. As such, it controls the early stages of mammalian embryogenesis, functions as a repressor of differentiation-specific genes and regulates the pluripotent downstream network in human embryonic stem cells (hESCs)^10,11^. The OCT4B transcript variant contains exon 1b, and exons 2, 3, 4 and 5. It is primarily expressed in the cytoplasm of cancer cells and somatic cells^3,4^. The N-terminal domain of OCT4B has an inhibitory effect on the DNA-binding domain such that, in contrast to OCT4A, it cannot regulate nuclear gene transcription to sustain the pluripotent state^12,13^. The OCT4B variant can produce three protein isoforms by alternative translation initiation, i.e. OCT4B-265 (30 kDa), OCT4B-190 (23 kDa) and OCT4B-164 (20 kDa)^14^. The OCT4B1 transcript variant, which contains exon 1b, exon 2, exon 2b (which corresponds to the whole 225-nt intron 2 of the OCT4 sequence) and exons 3, 4 and 5^4^. It is expressed primarily in hESCs and embryonic carcinoma cells and is downregulated upon induction of differentiation. The OCT4B1 transcript can generate the three protein isoforms of OCT4B^15^. The OCT4B2 transcript variant is composed of exon 1b lacking a 630-nt fragment, 2, 2b, 3, 4 and 5^5^. This transcript is highly expressed in undifferentiated cells and is elevated under heat shock induction^5^. The OCT4B3 transcript variant, which is composed of exons 1b lacking a 207-nt fragment, exon 2, 2b, 3, 4 and 5, is expressed in various cancer cell lines^6^. The OCTB4 transcript variant, which is composed of exon 1b, exon 2 lacking a 28-nt fragment, exons 3, 4 and 5. It is expressed in various human pluripotent stem cells where it may play a role in the regulation of the cell cycle in pluripotent stem cells and is downregulated upon induction of differentiation^7^.

Eight OCT4 pseudogenes have also been identified, all displaying high homology to OCT4A^8^, and with several of them being transcribed and translated into proteins in various cancer cell lines and tissues^16,17^. Pseudogene 1 (1080 bp) is encoded by OCT4-Pg1 on chromosome 8, pseudogene 3 (1081 bp) is encoded by OCT4-Pg3 on chromosome 12 and pseudogene 4 (1083 bp) is encoded by OCT4-Pg4 on chromosome 1^8^. During differentiation of hESCs, OCT4A expression is silenced through Dnmt3a/3b-dependent methylation of its promoter and may be replaced by the expression of pseudogenes^8^.

Ectopic expression of OCT4A alone is sufficient to revert human fetal neural stem cells^18^, human primary keratinocytes^19^ and human amniotic fluid stem cells (hAFSCs)^20,21^ to functional pluripotency. For example, we previously showed that the reactivation of OCT4A expression in hAFSCs cultivated in hESC-like conditions is sufficient to progressively revert the cells to the pluripotent state, as evidenced by their ability to form well-differentiated teratomas^20,21^. First described by Prusa *et al*. in 2003, hAFSCs were later identified as a stem cell type of choice for regenerative applications, due to their fast growth kinetics, small size, and long telomeres^22,23^. Sharing similar features with other tissue-derived fetal stem cells^24,25^, hAFSCs^26^ can be easily isolated and expanded from mid-trimester amniotic fluid obtained by routine amniocentesis or from term amniotic fluid obtained at delivery without ethical restriction. However, a number of reports define undifferentiated hAFSCs cultivated in non-hESC-like conditions as sharing features of hESCs and expressing OCT4A^27,28^. If correct, this potentially hinders the clinical use of primary undifferentiated hAFSCs and could render the cells capable of behaving unpredictably and with harm if transplanted *in vivo* in their undifferentiated state. It is therefore of paramount importance to carefully examine the expression of OCT4A in hAFSCs^14^.

Here, we present a systematic review of the literature to investigate whether published studies of hAFSCs distinguished OCT4A from other OCT4 isoforms. Our findings suggest that previous reports of OCT4A expression in hAFSCs may be due to cross-reaction with other isoforms and/or to a non-specific signal. Using reverse transcription-polymerase chain reaction (RT-PCR), immunocytochemistry and western blotting, we were unable to detect any population of OCT4A+ cells existing within the primary hAFSC population. The findings reported below therefore confirm that hAFSCs, either fresh or frozen, do not express OCT4A.

## Results

### Systematic review of studies on OCT4A in hAFSCs

OCT4A expression in hAFSCs is a subject of controversy and we believe that paying careful attention when designing primers should clarify this. Since exon 1 is unique to the OCT4A transcript, the forward primer should lie in exon 1 when detecting gene expression using RT-PCR (Fig. 1, Supplementary Fig. 1a), as recommended by Wang *et al*.^14^.

The criteria for OCT4A expression includes nuclear but not cytoplasmic localization by immunohistochemistry using antibodies specific for OCT4A, a molecular weight of 48–53 kDa, use of primers that do not amplify any of the pseudogenes for RT-PCR, and use of appropriate positive (pluripotent cells) and negative (differentiated cells or cell line expressing OCT4 transgene such as HEK293T, HeLa and HepG2 cell lines) controls for western blotting, flow cytometry, RT-PCR and immunohistochemistry.

Our systematic review, last updated on 19.11.2018, combined searches from the Web of Science and MEDLINE and found 1873 publications, 488 of which were duplicates and 1286 were non-relevant papers. Out of the 114 publications left, 5 were literature reviews, 17 publications were about cells from fetal tissue only, 6 were not in not written in English and 27 were about cellular reprogramming. The remaining 59 publications included 51 reporting positive RT-PCR results for OCT4 (only 45 gave primers information), 6 used flow cytometry and 20 used immunocytochemistry (19 gave antibody information) (Table 1).

**Table 1:** List of references reporting a positive expression in hAFSCs.

	c-Kit	RT-PCR	FC		IF			WB			PMID
				C		C	CL		C	S	
1	ND	Y	N	—	N	—	—	N	—	—	24480362
2	ND	Y	N	—	N	—	—	N	—	—	27434028
3	ND	Y	N	—	N	—	—	N	—	—	22916064
4	ND	Y	N	—	N	—		N	—	—	29573382
5	ND	Y	N	—	N	—	—	N	—	—	16617328
6	ND	Y	N	—	N	Y	Cyt				22377907
7	Y	Y	N	—	N	—	—	N	—	—	
8	ND	Y	N	—	N	—	—	Y	Y	Y	24647685
9	Y	Y	N	—	Y	N	Cyt	Y	—	—	24571984
10	Y	Y	N	—	N	—		N	—	—	22649611
11	Y	Y	N	—	Y	N	—	N	—	—	24101862
12	ND	Y	N	—	N	—	—	N	—	—	22665987
13	Y	Y	N	—	Y	N	Nuc	N	—	—	20955626
14	Y	Y	N	—	N	—	—	N	—	—	20939691
15	ND	Y	N	—	N	Y	Cyt	N	—	—	26720151
16	ND	Y	N	—	N	—	—	N	N	N	22530853
17	ND	Y	N	—	N	—	—	N	—	—	29688163
18	ND	Y	N	—	N	—	—	N	—	—	27434028
19	ND	Y	N	—	N	—	—	N	N	N	27818691
20	ND	N	N	—	Y	N	—	N	—	—	18564037
21	ND	Y	N	—	N	—	—	N	—	—	21774692
22	ND	Y	N	—	N	—	—	N	—	—	23806299
23	ND	Y	N	—	N	—	—	N	—	—	18062170
24	ND	Y	Y	—	N	Y	Cyt	N	—	—	28854517
25	ND	Y	N	—	Y	N	Nuc	Y	—	—	20221716
26	ND	N	Y	—	N	—	—	N	—	—	27465073
27	ND	Y	Y	—	Y	N	Nuc	N	—	—	18047393
28	ND	Y	N	—	N	—	—	N	—	—	17227297
29	ND	Y	N	—	N	—	—	N	—	—	16617328
30	ND	N	N	—	N	—	—	N	—	—	0123350
31	ND	Y	N	—	N	—	—	N	—	—	22200372
32	Y	Y	Y	N	Y	N	Cyt	N	—	—	23050522
33	ND	N	Y	N	N	—	—	N	—	—	27240324
34	ND	Y	N	—	Y	N	Cyt	Y	N	—	12832377
35	ND	Y	N	—	N	—	—	N	—	—	2019498
36	ND	Y	N	—	N	—	—	N	—	—	28379622
37	ND	Y	N	—	Y	Y	Cyt	N	—	—	6306422
38	ND	Y	N	—	Y	Y	Cyt	N	—	—	15105397
39	ND	Y	N	—	N	—	—	N	—	—	20708517
40	Y	Y	N	—	Y	Y	Cyt	Y	N	—	18760782
41	Y	N	N	—	Y	N	Nuc	Y	N	Nuc	24788911
42	N	N	N	—	Y	N	Nuc + C	N	—	—	26712153
43	ND	Y	—	—	Y	N	Nuc + Cyt	N	—	—	28672915
44	ND	N	Y	—	Y	N	Nuc + Cyt	N	—	—	23326421
45	ND	Y	N	—	Y	N	—	N	—	—	16555279
46	ND	Y	N	—	Y	N	—	N	—	—	20708517
47	ND	Y	N	—	N	—	—	N	—	—	17227297
48	ND	Y	N	—	N	—	—	N	—	—	21459439
49	ND	Y	N	—	N	—	—	N	—	—	18062170
50	ND	Y	N	—	Y	N	Nuc	N	—	—	19215679
51	ND	Y	N	—	N	—	—	N	—	—	25880317
52	ND	Y	N	—	N	—	—	Y	N	—	24375948
54	ND	Y	N	—	N	—	—	N	—	—	27803714
55	ND	Y	N	—	N	—	—	Y	N	—	25385323
56	ND	Y	N	—	N	—	—	Y	N	—	24798073
57	ND	Y	N	—	Y	—	—	N	—	—	18569033
58	ND	Y	N	—	Y	N	Cyt	N	—	—	18023443
59	ND	Y	N	—	N	—	—	N	—	—	25319435

PM: promoter methylation; RT: RT-qPCR; FC: flow cytometry; IF: immunofluorescence; CL: cellular localisation; Nuc: nuclear; Cyt: cytoplasmic; WB: western blotting; C: study includes appropriate controls; S: protein size indicated; N: no; Y: yes; ND: not determined.

Table 2 lists the primers used in the studies identified. Of these, 27 did not use a forward primer lying in exon 1, which is uniquely contained in the 5′ sequence of the OCT4A transcript, but not in the OCT4B and OCT4B1 transcript variants (primer sets 1,3,5,6,7,8,0,10,12,13,16,18,19,21,22,23,24,25,26,27,28,30 and 31 (Table 2)). Out of the 21 papers using a forward primer lying in exon 1, 20 used primers that also bind to pseudogene sequences (OCT4-Pg1 and OCT4-Pg3 for primer set 2; OCT4-Pg1 only for prier set 4,15,17 and 29; OCT4-Pg1, OCT4-Pg3 and OCT4-Pg4 for primer set 11 and 14) whilst only one publication (PMID 24375948) used primers that detects OCT4A exclusively (primer set 20). Despite this, appropriate positive and negative controls must be used to avoid potential bias. This should be performed in conjunction with the sequence of the amplification product to confirm detection of the isoform OCT4A.

**Table 2:** List of primers used in the references listed in Table 1.

PMID	Primer set	Primer sequence	Forward primer specificity	Reverse primer specificity	Size bp
27434028	Set 1	F: CCATGCATTCAAACTGAGGT R: CCTTTGTGTTCCCAATTCCT	OCT4A + B OCT4-Pg1	OCT4A + B	146 bp
29688163	Set 1	F: CCATGCATTCAAACTGAGGT R: CCTTTGTGTTCCCAATTCCT	OCT4A + B OCT4-Pg1	OCT4A + B	146 bp
22665987	Set 2	F: CGTGAAGCTGGAGAAGGAGAAGCTG R: CAAGGGCCGCAGCTTACACATGTTC	OCT4A OCT4-Pg1 OCT4-Pg3	OCT4A OCT4-Pg1 OCT4-Pg3	247 bp
0123350	Set 2	F: CGTGAAGCTGGAGAAGGAGAAGCTG R: CAAGGGCCGCAGCTTACACATGTTC	OCT4A OCT4-Pg1 OCT4-Pg3	OCT4A OCT4-Pg1 OCT4-Pg3	247 bp
16306422	Set 2	F: CGTGAAGCTGGAGAAGGAGAAGCTG R: CAAGGGCCGCAGCTTACACATGTTC	OCT4A OCT4-Pg1 OCT4-Pg3	OCT4A OCT4-Pg1 OCT4-Pg3	247 bp
15105397	Set 2	F: CGTGAAGCTGGAGAAGGAGAAGCTG R: CAAGGGCCGCAGCTTACACATGTTC	OCT4A OCT4-Pg1 OCT4-Pg3	OCT4A OCT4-Pg1 OCT4-Pg3	247 bp
24571984	Set 2	F: CGTGAAGCTGGAGAAGGAGAAGCTG R: CAAGGGCCGCAGCTTACACATGTTC	OCT4A OCT4-Pg1 OCT4-Pg3	OCT4A OCT4-Pg1 OCT4-Pg3	247 bp
16555279	Set 2	F: CGTGAAGCTGGAGAAGGAGAAGCTG R: CAAGGGCCGCAGCTTACACATGTTC	OCT4A OCT4-Pg1 OCT4-Pg3	OCT4A OCT4-Pg1 OCT4-Pg3	247 bp
20708517	Set 2	F: CGTGAAGCTGGAGAAGGAGAAGCTG R: CAAGGGCCGCAGCTTACACATGTTC	OCT4A OCT4-Pg1 OCT4-Pg3	OCT4A OCT4-Pg1 OCT4-Pg3	247 bp
17227297	Set 2	F: CGTGAAGCTGGAGAAGGAGAAGCTG R: CAAGGGCCGCAGCTTACACATGTTC	OCT4A OCT4-Pg1 OCT4-Pg3	OCT4A OCT4-Pg1 OCT4-Pg3	247 bp
21459439	Set 2	F: CGTGAAGCTGGAGAAGGAGAAGCTG R: CAAGGGCCGCAGCTTACACATGTTC	OCT4A OCT4-Pg1 OCT4-Pg3	OCT4A OCT4-Pg1 OCT4-Pg3	247 bp
18062170	Set 2	F: CGTGAAGCTGGAGAAGGAGAAGCTG R: CAAGGGCCGCAGCTTACACATGTTC	OCT4A OCT4-Pg1 OCT4-Pg3	OCT4A OCT4-Pg1 OCT4-Pg3	247 bp
19215679	Set 2	F: CGTGAAGCTGGAGAAGGAGAAGCTG R: CAAGGGCCGCAGCTTACACATGTTC	OCT4A OCT4-Pg1 OCT4-Pg3	OCT4A OCT4-Pg1 OCT4-Pg3	247 bp
20955626	Set 2	F: CGTGAAGCTGGAGAAGGAGAAGCTG R: CAAGGGCCGCAGCTTACACATGTTC	OCT4A OCT4-Pg1 OCT4-Pg3	OCT4A OCT4-Pg1 OCT4-Pg3	247 bp
24647685	Set 2	F: CGTGAAGCTGGAGAAGGAGAAGCTG R: CAAGGGCCGCAGCTTACACATGTTC	OCT4A OCT4-Pg1 OCT4-Pg3	OCT4A OCT4-Pg1 OCT4-Pg3	247 bp
25880317	Set 2	F: CGTGAAGCTGGAGAAGGAGAAGCTG R: CAAGGGCCGCAGCTTACACATGTTC	OCT4A OCT4-Pg1 OCT4-Pg3	OCT4A OCT4-Pg1 OCT4-Pg3	247 bp
16617328	Set 3	F: ACATGTGTAAGCTGCGGCCR: GTTGTGCATAGTCGCTGCTTG	OCT4A + B OCT4-Pg1 OCT4-Pg4	OCT4A + B	
22377907	Set 4	F: CTGTAACCGGCGCCAGAA R:TGCATGGGAGAGCCCAGA	OCT4A OCT-Pg1	OCT4A + B	240 bp
22649611	Set 5	F: CGACCATCTGCCGCTTTGAG R: CCCCCTGTCCCCCATTCCTA	OCT4A + B OCT4-Pg3 OCT4-Pg4	OCT4A + B	
23050522	Set 6	F:TCGAGAACCGAGTGAGAGGC R: CACACTCGGACCACATCCTTC	OCT4A + B OCT4-Pg1	OCT4A + B	
12832377	Set 7	F: GACAACAATGAAAATCTTCAGGAGA R: TTCTGGCGCCGGTTACAGAACCA	OCT4A + B OCT4-Pg4	OCT4A + B	
2019498	Set 8	F: CGAGAAGGATGTGGTCCGAG R: CAGAGGAAAGGACACTGGTC	OCT4A + B OCT4-Pg1 OCT4-Pg4	OCT4A + B	
28379622	Set 9	F: CTTCAATCGCATATTCTTTAACCA R: GGAGGAAGCTGA CAACAACG	OCT4A + B OCT4-Pg3	OCT4A + B	
28672915	Set 10	F: GTGGAGGAAGCTGACAACAA R: TCTCCAGGTTGCCTCTCACT	OCT4A + B OCT4-Pg1 OCT4-Pg3 OCT4-Pg4	OCT4A + B	118 bp
23326421	Set 11	F: CAATTTGCCAAGCTCCTGA R: CAGATGGTCTTTGGCTGAAC	OCT4-Pg1 OCT4-Pg3 OCT4-Pg4 OCT4A	OCT4A + B	
27803714	Set 12	F: CGAGAAGGATGTGGTCCGAG R: CAGAGGAAAGGACACTGGTC	OCT4A + B OCT4-Pg1	OCT4A + B	
18047393	Set 13	Applied Biosystems Hs00742896_s1	OCT4A + B OCT4Pg4 OCT4Pg1	OCT4A + B OCT4Pg4 OCT4Pg1	
25385323	Set 14	F: GGCTTGGAGACCTCTCAGCCTG R: TGCAGCAAGGGCCGCAGCTTAC	OCT4A OCT4-Pg1 OCT4-Pg3 OCT4-Pg4	OCT4A OCT4-Pg1 OCT4-Pg4	247 bp
24798073	Set 15	F: GATGGCGTACTGTGGGCCC R: TGGGACTCCTCCGGGTTTTG	OCT4A OCT4-Pg1	OCT4A OCT4-Pg1	
24101862	Set 16	F: CTCACCCTGGGGGTTCTAT R: CTCCAGGTTGCCTCTCACTC	OCT4A + B OCT4-Pg3	OCT4A + B	
18569033	Set 17	F: CAGGAGATATGCAAAGCAGAA R: AGCCTCAAAATCCTCTCGTT	OCT4-Pg1 OCT4A	OCT4A + B	
18023443	Set 18	F: GAGGAAGCTGACAACAATGAA R: GGTTTTCTTTCCCTAGCTCCT F: CAGGAGATATGCAAAGCAGAA R: AGCCTCAAAATCCTCTCGTT	OCT4A + B OCT4-Pg3	OCT4A + B	
25319435	Set 19	F: GAAGGATGTGGTCCGAGTGT R: GTGAAGTGTGAGGGCTCCCATA	OCT4A + B OCT4-Pg1 OCT4-Pg3	OCT4A + B	
24375948	Set 20	F: TCCCTTCGCAAGCCCTCAT R: TGACGGTGCAGGGCTCCGGGGAGG	OCT4A	OCT4A	
23326421	Set 21	F: CAATTTGCCAAGCTCCTGA R: CAGATGGTCTTTGGCTGAAC	OCT4A + B OCT4-Pg3 OCT4-Pg4 OCT4-Pg1	OCT4A + B	
28672915	Set 22	F: GTGGAGGAAGCTGACAACAA R: TCTCCAGGTTGCCTCTCACT	OCT4A + B OCT4-Pg3 OCT4-Pg4 OCT4-Pg1	OCT4A + B	118 bp
23806299	Set 23	F: GTTCCCAATTCCTTCCTTA R: TAAGGAAGGAATTGGGAAC	OCT4A + B	OCT4A + B	167 bp
27818691	Set 24	F: TATCGAGAACCGAGTGAGAG R: TACAGTGCAGTGAAGTGAGG	OCT4A + B	OCT4A + B	294 bp
22530853	Set 25	F: ATCAAGCAGCGACTATGCAC R: GAAAGGGACCGAGGAGTACA	OCT4A + B	OCT4A + B	
26720151	Set 26	F: GAGGAGTCCCAGGACATGAA R: GTGGTCTGGCTGAACACCTT	OCT4A + B	OCT4A + B	151 bp
20939691	Set 27	F: GCCTCCAAACAACCTTAGCA R: GCTGGGCTCCAGATAGACAC	OCT4A + B	OCT4A + B	478 bp
22649611	Set 28	F: CGACCATCTGCCGCTTTGAG R: CCCCCTGTCCCCCATTCCTA	OCT4A + B OCT4-Pg3 OCT4-Pg4	OCT4A + B	240 bp
22377907	Set 29	F: CTGTAACCGGCGCCAGAA R:TGCATGGGAGAGCCCAGA	OCT4A OCT-Pg1	OCT4A + B	
16617328	Set 30	F: ACATGTGTAAGCTGCGGCC R: GTTGTGCATAGTCGCTGCTTG	OCT4A + B OCT4-Pg1 OCT4-Pg4	OCT4A + B	
24480362	Set 31	F:GACAGGGGGAGGGGAGAGCTAGG R:CTTCCCTCCAACCAGTTGCCCCAAAC	OCT4A + B	OCT4A + B	144 bp

Y: yes, N: no, C: study includes appropriate control.

A total of 20 publications reported OCT4 expression using specific antibodies against OCT4 for either flow cytometry, western blotting and/or immunofluorescence applications (listed Table 1, antibodies used listed in Table 3). The description of the methodology from these studies was deemed insufficient during the systematic review. For example, the antibodies used were not always specified, positive and negative controls were not systematically included to confirm antibody specificity, fluorescence was not exclusively present in the nucleus. No study used controls to confirm exclusive nuclear staining and OCT4A-specificicity, for example using a cell line expressing pseudogene OCT4-Pg1. This is particularly important for western blotting since the OCT4A protein has the same molecular weight (39 kDa) as OCT4-Pg1^8^. Similarly, primers amplifying both OCT4A and OCT4-Pg1 (Table 2, primer sets 3, 4, 6, 7, 14, 16 and 18) do not allow determination of OCT4 expression, especially in the absence of immunostaining to confirm nuclear localization. Thus, we concluded that given our current state of knowledge and list of OCT4A expression criteria, positive OCT4A expression in hAFSCs cultivated in non-pluripotent conditions remained inconclusive in all studies listed in Table 1.

**Table 3:** List of antibodies used in the references listed in Table 1.

Method used for detection	Manufacturer	Antibody Product Number	PMID
IF	R&D system	AF1759	22377907
WB	Abcam	NG	24647685
WB	Cell Signaling	2890	24571984
IF	Beckton Dickinson, NJ	NG	24101862
IF	Santa Cruz Biotechnology	NG	20955626
IF	Abcam	AB19857	26720151
FC	Santa Cruz Biotechnology	NG	18564037
FC IF	Affymatrix Santa Cruz Biotechnology	E660 Sc-9081	28854517
WB IF	Santa Cruz Biotechnology	Sc-5279	20221716
FC, IF	Santa Cruz Biotechnology	NG	18047393
IF FC	Santa Cruz Biotechnology R&D system	Sc-5279 AF1759	23050522
WB, IF	Santa Cruz Biotechnology	Sc-5279	12832377
IF	Santa Cruz Biotechnology	Sc-5279	16306422
IF	Santa Cruz Biotechnology	Sc-5279	15105397
WB. IF	Cell Signaling	2890	18760782
WB, IF	Merck Millipore	AB3209	24788911
IF	Bioss	bsm-52001M	26712153
IF	Cell Signaling	D7O5Z	28672915
FC	BD Biosciences	NG	27465073
IF	Abcam Cell Signaling	AB19857 2750	23326421
IF	Not given		16555279
IF	Chemicon, Temecula	NG	20708517
IF	Santa Cruz Biotechnology	Sc-5279	19215679
WB	Santa Cruz Biotechnology	NG	24375948

IF: immunofluorescence; WB: western blotting. NG: not given.

### Validation of the specificity of OCT4 antibodies to detect the OCT4A isoform immunofluorescence

To examine the specificity of OCT4 antibodies for the OCT4A isoform, we selected the eight antibodies most commonly used to assess OCT4A isoform expression in the literature (listed in Table 4). We used hESCs as positive control since OCT4A is expressed in human pluripotent stem cells. The osteoblast cell line MG63 cells and hAFSCs differentiated down the osteogenic lineage for 3 weeks (Diff cells) were selected as negative controls because OCT4A is downregulated upon differentiation. To confirm OCT4A specificity and the absence of OCT4 pseudogene expression, we used HeLa, HEK293T (293T) and HepG2 cell lines which express pseudogenes Pg1, Pg3 and Pg4^29^, as additional negative controls. We used immunofluorescence, flow cytometry and/or western blotting to determine whether each antibody fulfilled the criteria for specific detection of OCT4A (nuclear localisation, the absence of cytoplasmic staining and a molecular weight of 48–53 kDa as opposed to 39–45 kDa for OCT4B).

**Table 4:** List of antibodies tested in this study.

	Supplier	Reference
1	Santa Cruz Biotechnology	Sc-5279
2	Santa Cruz Biotechnology	Sc-8629
3	Santa Cruz Biotechnology	Sc-9081
4	Miltenyi Biotechnology	130-105-606
5	Millipore	MAB4419
6	Abcam	Ab19857
7	R&D systems	MAB17591
8	R&D systems	IC1759P

**Figure 2:**
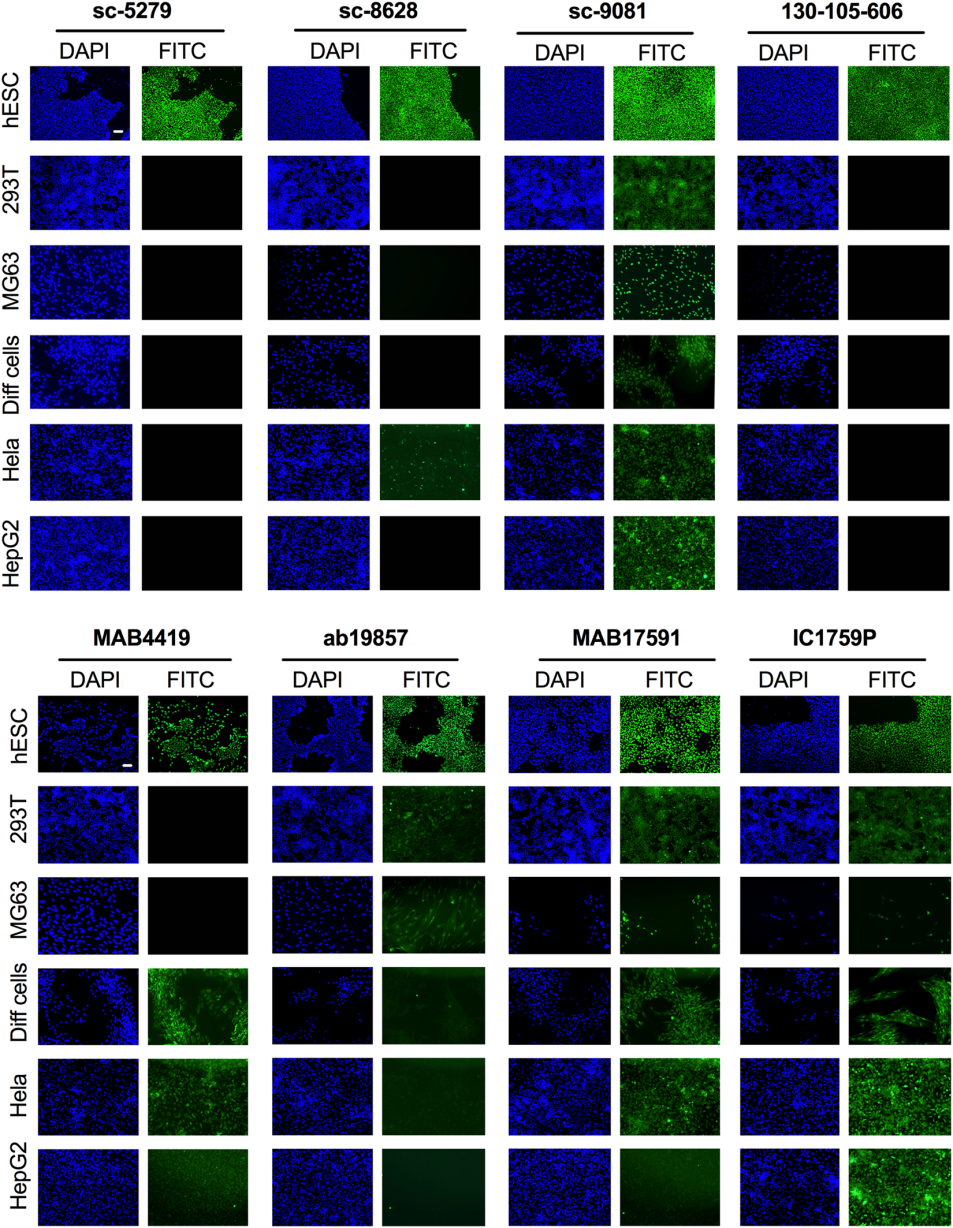
Specificity of various antibodies against OCT4A. Immunofluorescent cell staining showing expression of OCT4 in hESCs (positive control), MG63 (negative control), 293T, osteoblast-differentiated hAFSCs, HeLa cells and HepG2 cells. Nuclei were stained with DAPI (blue). Scale bar 50 μm.

We first tested the eight antibodies by immunofluorescence (IF) (Fig. 2). IF staining using sc-5279 and 130-105-606 showed positive nuclear localisation in the positive control hESCs and the absence of staining in all negative controls, confirming the specificity of these antibodies for the nuclear detection of the OCT4A isoform. IF staining with sc-8628 showed nuclear localisation in hESCs but also strong nuclear staining in a subset of HeLa cells, suggesting this antibody detects OCT-Pg1, 3 and/or 4. IF staining with sc-9081, MAB17591 and IC1759P showed positive nuclear staining of hESCs but also positive nuclear staining for all five negative controls, revealing the non-specificity of the antibody for the OCT4A isoform and its cross-reactivity with isoform OCT4B and pseudogenes OCT4-Pg1, 3 and/or 4. IF staining with MAB17591 also showed positive cytoplasmic staining in osteoblast-differentiated hAFSCs and IC1759P also showed positive cytoplasmic staining in HEK293T and osteoblast-differentiated hAFSCs. IF staining with MAB4419 showed positive nuclear staining of hESCs but also positive nuclear staining in HeLa cells and HepG2 cells, and positive cytoplasmic staining in osteoblast-differentiated hAFSCs, indicating the importance of including several negative control cell lines to test for cross-reactivity. IF staining with Ab19857 showed positive nuclear staining in hESCs but also strong positive nuclear and cytoplasmic staining of 293T and MG63 cells and faint cytoplasmic staining in osteoblast-differentiated hAFSCs, HeLa and HepG2 cells. In conclusion, our results indicate that only sc-5279 and 130-105-606 antibodies are suitable for the detection of OCT4A isoform by immunofluorescence.

The mid-trimester hAFSC population is composed of two subsets of cells that can be distinguished morphologically and immunophenotypically: CD117^+^CD105^+^CD90^+^CD73^+^ spindle-shaped cells (SS-hAFSCs) and CD117^+^CD105^−^CD90^−^CD73^+^round-shaped cells (RS-hAFSCs)^30^. The cells are routinely expanded on tissue culture treated dishes without feeders in culture medium used for multipotent stem cells, such as Dulbecco’s Modified Eagle’s Medium supplemented with 10% fetal bovine serum (D10) or in α-MEM Medium supplemented with 20% Chang Medium B, 2% Chang Medium C and 20% Fetal Bovine Serum (Chang) (Supplementary Fig. 2).

**Figure 3:**
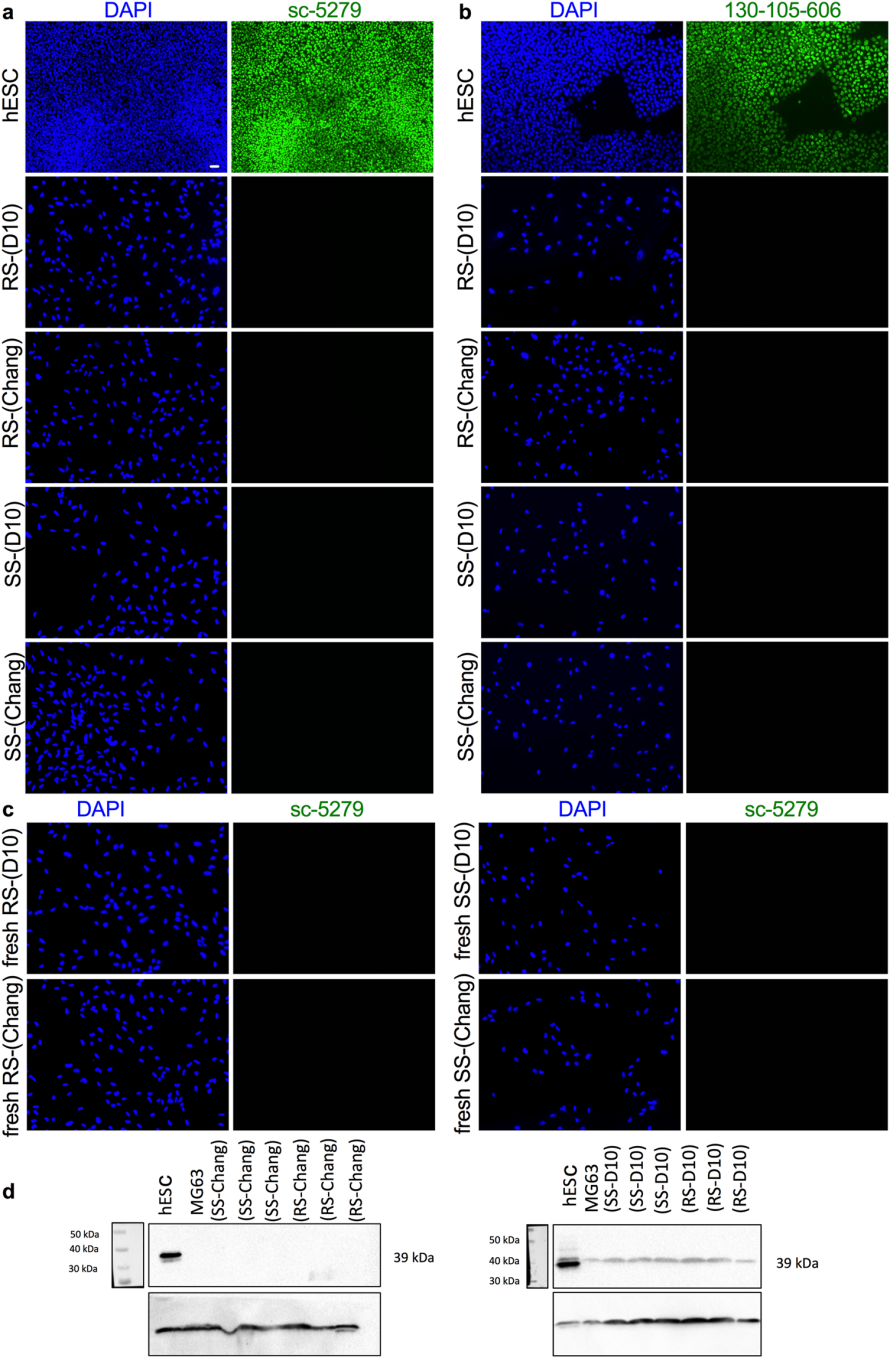
Expression of OCT4A in hAFSCs. Immunofluorescent cell staining showing expression of OCT4A using the antibodies sc-5279 (**a**) and 130-105-606 antibody (**b**) in hESCs (positive control) and RS-hAFSCs and SS-hAFSCs cultivated in Chang C or D10 culture medium that have previously been expanded, frozen and thawed or in freshly-isolated cells that have not been expanded beyond passage 1 and never frozen (**c**) (40X magnification). Nuclei were stained with DAPI (blue). Scale bar 50 μm. (**d**) Western blotting for OCT4A detection in RS-hAFSCs and SS-hAFSCs cultivated in Chang C or D10 culture medium and in hESCs (positive control) and MG63 (negative control). Cell lysates were prepared and western blot was performed using sc-5279 antibody against OCT4A and antibody against β actin.

We next used the antibodies sc-5279 and 130-105-606 to determine whether hAFSCs express OCT4A. Immunofluorescence revealed the absence of staining of both hAFSC subsets SS-hAFSCs and RS-hAFSCs cultivated either in Chang C or D10 medium (Fig. 3a,b). However, these cells have been previously expanded, frozen and thawed for analysis. We therefore hypothesized that OCT4 might be expressed in freshly-isolated cells and progressively lost during *ex vivo* expansion or that freshly-isolated populations contain a small number of cells expressing OCT4A that do not undergo clonal expansion. To test this hypothesis, we analysed freshly-isolated passage 1 SS-hAFSCs and RS-hAFSCs cultivated in either D10 or Chang culture medium immediately after isolation that had not been expanded in culture beyond the first passage. Results indicated the absence of staining using the sc-5279 antibody (Fig. 3c) and the 130-105-606 antibody (data not shown) in both cell subsets.

### Western blotting

As the sc-5279 antibody is suitable for western blot analysis, we next confirmed the expression of the OCT4A protein isoform in hESCs but its absence in the negative control MG63 cells and in freshly-isolated passage 1 SS-hAFSCs and RS-hAFSCs cultivated in D10 or Chang medium (Fig. 3d), with a faint non-specific band present in all cell lines (Fig. 3d).

### Flow cytometry

**Figure 4:**
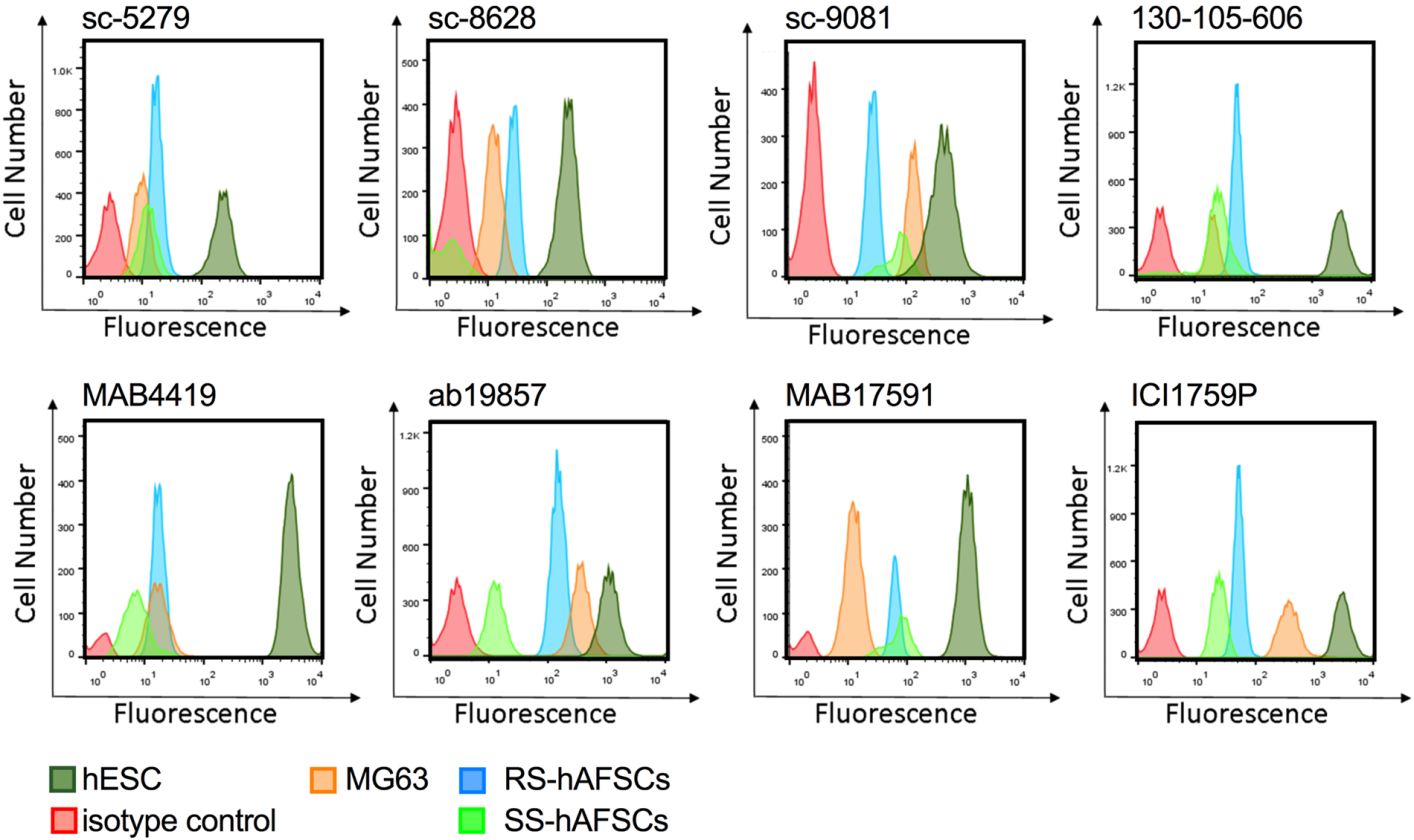
Flow cytometry analysis of hAFSCs. Flow cytometry showing OCT4 expression in hESCs (dark green tracing), MG63 (yellow tracing), RS-hAFSCs (blue tracing) and SS-hAFSC (light green tracing) using the antibodies shown. The red tracing shows the primary antibody only control.

We next used flow cytometry to confirm the results obtained using immunofluorescence. We tested the eight different antibodies listed in Table 4, with hESCs as positive control and MG63 cells as negative control. Results showed positive expression in hESCs for all antibodies (Fig. 4). For all antibodies, the peak of fluorescence obtained for the negative control MG63 was distinct from the peak corresponding to the primary antibody-only control, indicating that autofluorescence could be interpreted as false-positive in the absence of positive controls.

### hAFSCs do not express most pluripotency markers

**Figure 5:**
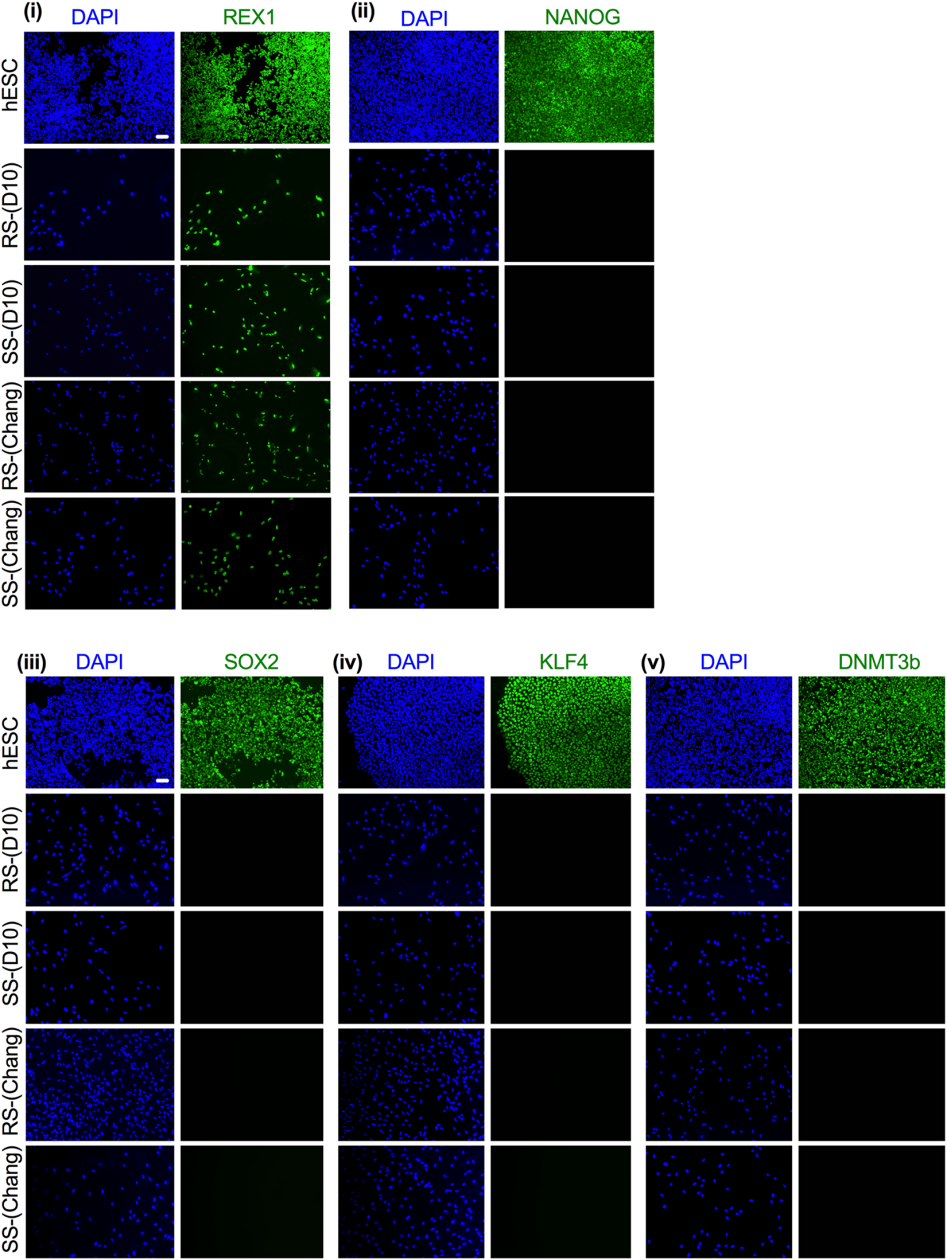
Expression of pluripotency markers in hAFSCs. Immunofluorescence showing expression of the pluripotency associated markers REX1, NANOG, SOX2, KLF4 and DNMT3b. Nuclei were stained with DAPI (blue). Scale bar 50 μm.

Since the nuclear OCT4A isoform is exclusively expressed in pluripotent cells, we first assessed the expression of other pluripotency-associated markers in SS-hAFSCs and RS-hAFSCs cultivated either in D10 or Chang medium. We found that REX1 was present in the nucleus of both cell subsets in either culture medium. However, NANOG, SOX2, KLF4 and DNMT3b were only expressed in the positive control (hESCs) but not in hAFSCs cultivated either in D10 or Chang medium (Fig. 5), confirming that both SS and RS hAFSCs do not express pluripotency-associated markers except REX1.

### hAFSCs express transcripts for OCT4-Pg1 and OCT4-Pg4 but not OCT4A

**Figure 6:**
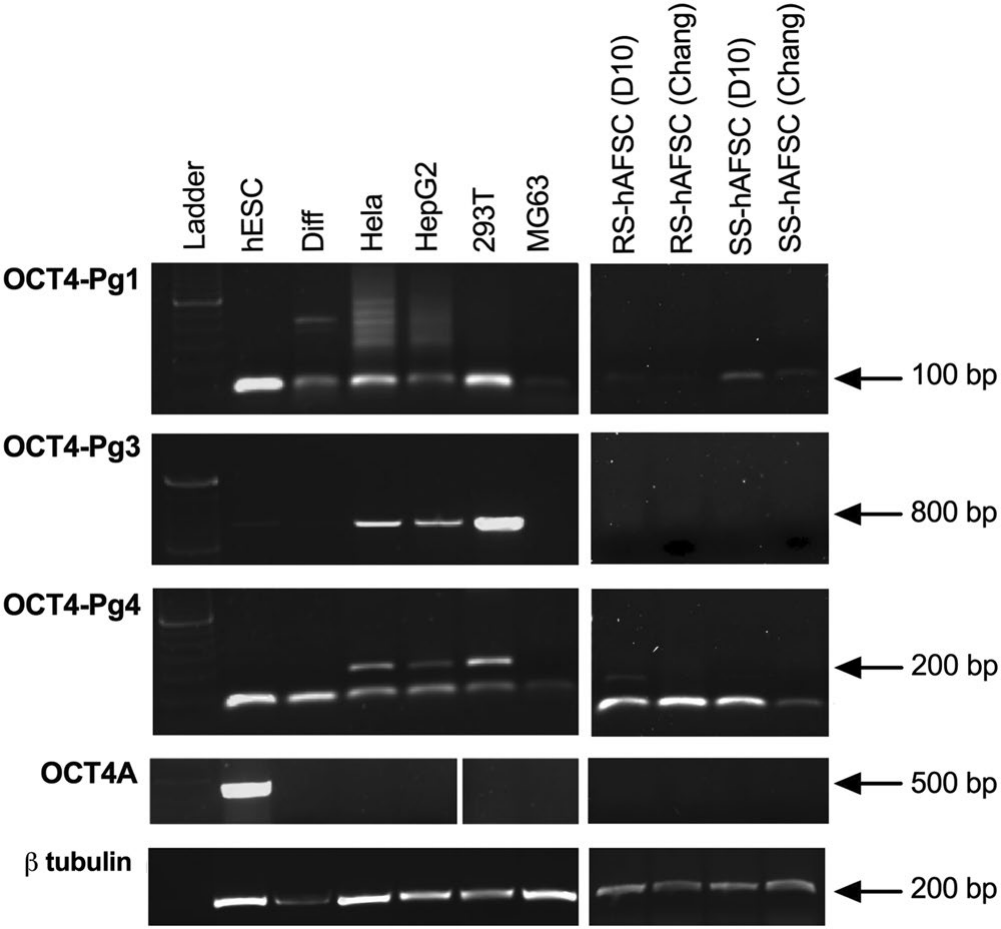
RT-PCR analysis for the housekeeping genes βtubulin, OCTA4-Pg1, OCT4-Pg3, OCT4-Pg4 and OCT4A expression in hESCs, differentiated cells, HeLa, HEPG2, 293T, MG63, and RS-hAFSCs and SS-hAFSCs cultivated in D10 or Chang medium. The image was overexposed to ensure that even faint bands were visible. Overexposure is visible in red on the image.

Although OCT4A protein was not detected in freshly-isolated hAFSCs, there is a possibility that the gene was transcribed but not translated. To test this hypothesis, we used primers specifically amplifying *OCT4A, OCT4A-Pg1, OCT4A-Pg3* and *OCT4A-Pg4* sequences (sequences in Supplementary Fig. 1b) and assessed gene expression in hESCs (positive control for OCT4A), osteoblast-differentiated hAFSCs and MG63 cells (negative control for OCT4A), HeLa, HepG2, and 293T (positive control for *OCT4A-Pg1, OCT4A-Pg3* and *OCT4A-Pg4*). Results confirmed the expression of *OCT4A-Pg1*, *OCT4A-Pg3* and *OCT4A-Pg4* in HeLa, HepG2, and 293T, as well as expression of *OCT4A-Pg1* in hESCs, osteoblast-differentiated hAFSCs and MG63 cells and the expression of *OCT4A-Pg3* in hAFSCs and in osteoblast-differentiated hAFSCs (Fig. 6). We also confirmed the expression of the OCT4A isoform in hESCs and its absence in all negative control cell lines. Finally, our results confirmed that OCT4A is not transcribed in freshly-isolated (never frozen) hAFSCs, but that *OCT4A-Pg1* and *OCT4A-Pg4* are transcribed in these populations, which could somehow be related to their plasticity and their ability to reach pluripotency without any genetic manipulation^31^. This may further explain the reports of positive OCT4A expression in hAFSCs.

## Methods

### Systematic review

A Medline® and Web of Science™ search of journal articles. Our MEDLINE Ovid search strategy was: (1) amniotic fluid/; (2) fetal stem cells/or mesenchymal stromal cells/; (3) (amniotic fluid or amniotic stem cell* or fetal stem cell* or mesenchymal stem cell* or amniocytes* or MSC*; (4) 1 or 2 or 3; (5) octamer Transcription Factor-3; (6) (pou domain class 5 transcription factor 1 or POU5F1 or OCT4 or octamer transcription factor 4 or oct 4 or oct 3 or oct3 or “oct3?4” or octamer transcription factor 3); (7) 5 or 6; (8) 4 and 7; (9) humans/; (10) human*, (11) 9 or 10; (12) 8 and 11. Full text copies of relevant articles were retrieved and read in full; references were managed using Paper 3 for Mac software. Appropriate controls included pluripotent cells for positive controls and differentiated stem cells and cell lines expressing OCT4 transgenes for negative controls.

### Ethics

Amniotic fluid (AF) was collected from healthy donors after written informed consent was obtained from all participants or their legal guardians, in compliance with the Declaration of Helsinski. The ethical approval was given by the Research Ethics Committees of Hammersmith & Queen Charlotte’s Hospitals (2001/6234) for frozen samples and from NRES Committee London, Bloomsbury (14/LO/0863) for fresh samples, in compliance with UK national guidelines (Review of the Guidance on the Research Use of Fetuses and Fetal Material (1989)) also known as Polkinghorne Guildeline. London: Her Majesty’s Stationery Office, 1989: Cm762) for the collection of human fetal tissue for research.

### Cell isolation and culture

AF was collected from six different donors as a routine procedure during the mid-trimester (15–18 weeks of gestation) from pregnant women undergoing prenatal diagnosis for possible chromosomal abnormalities. Each AF sample was immediately centrifuged before the cell pellet was resuspended into a single cell suspension in StemMACS expansion media and seeded under xeno- and serum-free conditions at low density (10^2^ cells/cm^2^) onto a plastic culture dish. The cell cultures were then allowed to expand until clones >50 cells formed. Some clones contained round-shaped cells and others spindle-shaped cells. Clones presenting similar morphology were pooled, centrifuged and resuspended into a single cell suspension that was plated (at 10^4^ cells/cm^2^) on plastic culture dishes without feeders either in Dulbecco’s Modified Eagle’s Medium (DMEM-HG) (Invitrogen) supplemented with 10% fetal bovine serum (FBS) (Biosera), 2 mM L-Glutamine, 50 IU/ml penicillin and 50 mg/ml streptomycin (Gibco-BRL), referred to as D10, or in α-MEM Medium [Gibco/BRL] supplemented with 20% Chang Medium B [Irvine Scientific] and 2% Chang Medium C [Irvine Scientific], 20% Fetal Bovine Serum [Gibco/BRL], 1% L-Glutamine [Gibco/BRL], and 1% antibiotics (pen-strep) [Gibco/BRL], referred to as Chang C, at 37 C in 5% CO_2_. We analysed a total of three SS-hAFSCs and three RS-hAFSCs samples. All samples had normal karyotype and were used between passage 5–10 for frozen samples and at passage 1 for fresh samples. The hESC line H1 (WiCell Research Institute) was cultured in feeder-free conditions on Matrigel-coated plates in mTeSR (STEMCELL Technologies). The MG63 osteosarcoma cell line, liver hepatocellular carcinoma (HepG2) and HEK293T cells were cultured in D10 as described above. HeLa cell lines were obtained from the American type culture collection (ATCC, Manassas, USA) and cultured in D10.

### Osteogenic differentiation

Cells were seeded at 10,000 cells/cm^2^ in D10. When confluent, the expansion medium was replaced with freshly-prepared osteogenic medium (D10 supplemented with 0.2 mM ascorbic acid, 10 mM ß-glycerophosphate and 10^−8^ M dexamethasone (all Sigma-Aldrich)). Osteogenic medium was freshly prepared and replaced twice weekly for 21 days.

### Flow cytometry

Cells (n = 3 for SS-hAFSCs and n = 3 for RS-hAFSCs) were detached, washed in flow buffer (PBS + 3% BSA, Sigma) and centrifuged at 5000 g for 2 minutes before 1 × 10^5^ cells were resuspended in the appropriate primary antibody (listed in Table 3) at its optimal dilution (1:10) in flow buffer and incubated for 1 hour at 4 °C. For unconjugated antibodies, cells were then washed and resuspended in a 1:10 dilution of FITC-conjugated donkey anti-mouse (FITC conjugated donkey anti-rabbit IgG, FITC donkey anti-goat IgG and donkey anti-mouse IgG all from ImmunoResearch) for 30 minutes at 4 °C. Cells were then analysed using a Becton Dickinson FACScalibur flow cytometer (BD Biosciences) using Cell Quest Pro and FlowJo software.

### Immunofluorescence

Cells (n = 3 for SS-hAFSCs and n = 3 for RS-hAFSCs) were washed, fixed in 4% paraformaldehyde (PFA, Sigma) and permeabilized. Cells were then blocked for 30 min with blocking buffer (PBS supplemented with 2% bovine serum albumin (BSA) and 0.1% Tween) and incubated overnight in the dark with primary antibodies at their optimal dilution, i.e. SC-5279 (Santa Cruz, 1:200), SC-8628 (Santa Cruz, 1:200), SC-9081 (Santa Cruz, 1:200), 130-105-606 (Miltenyi Biotec, 1:100), MAB4419 (Millipore, 1:200), AB198579 (Abcam, 1:200), MAB17591 (R&D systems, 10ug/ml), IC1759P (R&D Systems, 10ug/ml), then washed and incubated with secondary antibody (Alexa Flour 488 Goat anti-rabbit IgG, Alexa Flour 488 Goat anti-mouse IgG, Alexa Flour 488 Donkey anti- goat IgG and Alexa Flour 488 Donkey anti- rat IgG (all from Invitrogen 1:500) for 1 hr at RT. Then counter-stained with 4′,6-diamidino-2-phenylindole (DAPI) and visualized immediately. Images were collected using a LeicaDM 6000 fluorescence microscope (40x PLAN APO objective) and transferred to Adobe Photoshop (Adobe Systems).

### Western blotting

Protein lysates (n = 3 for SS-hAFSCs and n = 3 for RS-hAFSCs) were generated using RIPA lysis buffer (150 mM sodium chloride, 1.0%Triton X-100, 0.5% sodium deoxycholate, 50 mMTris, pH 8.0, 1:100) containing 0.1% SDS. 25 μg of β-mercaptoethanol. Denatured lysates were then separated on an 8% -PAGE gel and blotted onto a Protran nitrocellulose transfer membrane (Whatman, Life Sciences). The membrane was blocked in 5% milk PBS-T (phosphate-buffered saline with 0.1% Tween-20) and immunoprobed with antibodies raised against different peptides containing primary antibody overnight at 4 °C: SC-5279 (Santa Cruz, 1:200), SC-8628 (Santa Cruz, 1:200), SC-9081 (Santa Cruz, 1:200), MAB4419 (Millipore, 1:200), AB198579 (Abcam, 1:200), MAB17591 (R&D systems, 0.5ug/ml), IC1759P (R&D Systems, 0.5ug/ml). The secondary antibodies used were Anti–mouse IgG HRP linked antibody (Cell signaling, 1:1000), Rabbit anti-rat HRP Conjugated (Thermo Fisher, 1:1000), Donkey anti-rabbit IgG HRP-linked (VWR, 1:1000), Donkey anti-goat IgG HRP-linked (Santa Cruz, 1:500). The loading control was β -actin (Abcam, 1:1000). The experiments were performed in triplicate.

### RT-PCR

Total RNA was extracted from 3–5 × 10^6^ cells (n = 3 for SS-hAFSCs and n = 3 for RS-hAFSCs) using the RNeasy Mini RNA kit (Qiagen) and cDNA was synthesised from 1 µg RNA using Pd(N)6 random hexamers (Amersham Pharmacia Biotech) and 1 ml of 200U M-MLV Reverse Transcriptase in the presence of dNTPs (Promega), according to the manufacturer’s instructions. The generated cDNA was amplified using the ABI StepOne Sequence Detector system (Applied Biosystems) and primers listed in Supplementary Fig. 1b. The results were then analysed by gel electrophoresis.

## Discussion

Mid-trimester amniotic fluid contains two sub-populations of cells that can be distinguished by their differential morphology and immunophenotype. The SS-hAFSC population has huge potential for regenerative medicine^27^. We, and others, have demonstrated therapeutic effects of these cells in mouse models of osteogenesis imperfecta^32^, hypoxic-ischemic encephalopathy^30^ or kidney damage^33,34^ for example. Moreover, they are currently under assessment in clinical trials targeting osteoarthritis, neuropathy and pelvic pain amongst others^35^. Conflicting reports exist regarding their expression of pluripotency markers. We establish here that the OCT4A isoform, which is exclusively present in the nucleus of pluripotent stem cells, is not expressed in human fetal stem cells isolated from mid-trimester amniotic fluid. Our results highlight the necessity of using appropriate positive controls not only for OCT4A but also for the OCT4 pseudogenes, as well as a combination of approaches to confirm the expression of OCT4A at the RNA and protein levels. With the advancement in the field there is now more robust techniques to address OCTA expression as established for other cell lines^28^. For immunostaining, flow cytometry and western blotting, the specificity of antibodies for OCT4A should be validated by positive expression in pluripotent cells and the absence of expression in multipotent cells, lineage differentiated cells and for cell lines expressing OCT4 pseudogenes. OCT4A protein should also be localized in the nucleus (as assessed by immunostaining) and should be the correct size (as determined by western blotting). For RT-PCR, it is necessary to use a forward primer located in OCT4A exon 1, primers that exhibit no specificity for the OCT4 pseudogene sequences, and to validate the specificity for OCT4A using positive and negative controls. To confirm positive expression, it would be strongly advised to sequence the amplification product, thus identifying it as OCT4A.

Critical examination of the studies identified by our systematic review and our robust hAFSC sample analysis led us to conclude that human amniotic fluid does not contain cells expressing OCT4A. However, it remains possible that some studies may have detected positive expression in some samples. Validating OCT4A specificity remains a technical challenge to unequivocally report the existence of a subset of cells present in the amniotic fluid expressing OCT4 or to document OCT4A reactivation upon *in vitro* expansion. For example, we previously reported that OCT4A expression in human mesenchymal stem cells (MSCs) from fetal-placental tissues was not attributable to different culture conditions, tissue sources, or gestational ages but instead to the techniques used^36^.

More recently, we showed that the human amniotic fluid contains MSCs of renal origin, and that the presence of these cells increases with gestational age^37^. Despite some cells expressing the pluripotency-associated markers TRA-1-60 and TRA-1-81, OCT4 protein was localized in the cytoplasm and absent in the nucleus.

The possibility that OCT4A expression occurs only in hAFSCs upon fresh isolation has led to the hypothesis that the amniotic fluid might contain a sub-population of pluripotent cells. However, the data from our laboratory suggest that this is not the case, as freshly-isolated cells seeded in culture media that do not maintain pluripotency (D10 and Chang C) failed to show OCT4A positivity. In addition, publications claiming OCT4A expression in hAFSCs have not demonstrated the role of OCT4 in maintaining self-renewal, as it has been done in hESCs using siRNAs^38,39^.

Our results do not allow us to conclude the absence of OCT4A-positive stem cells in the amniotic fluid. Instead, they confirm that OCT4A is not expressed in hAFSCs expanded *in vitro* in non-pluripotent conditions. Interestingly, we have reported that SS-hAFSCs may be more plastic than their postnatal counterparts since when they are cultivated in pluripotent conditions in the presence of valproic acid (VPA), they up-regulated OCT4A expression and reverted to functional *bona fide* pluripotency by reactivating the OCT4-downstream pluripotent pathway^27,31,40^. This suggests that despite OCT4A being downregulated, DNA conformation at the epigenetic level might be permissive to OCT4A reactivation. Moreover, it remains possible that samples isolated during the first trimester of pregnancy contain OCT4A^+^ cells. Our findings, if verified by reports of a lack of expression of additional pluripotency markers in hAFSCs along with their inability to form tumours in animal models despite being positive for REX1, could have important implications for the safe clinical use of mid-trimester hAFSCs.

## Supplementary information

Supplementary information
