# Publisher Correction: Human mid-trimester amniotic fluid (stem) cells lack expression of the pluripotency marker OCT4A

**DOI:** 10.1038/s41598-020-61555-5

**Published:** 2020-03-10

**Authors:** Filipa Vlahova, Kate E. Hawkins, Anna Maria Ranzoni, Kwan-Leong Hau, Rachel Sagar, Paolo De Coppi, Anna L. David, James Adjaye, Pascale V. Guillot

**Affiliations:** 1 University College London, Institute for Women’s Health, Maternal and Fetal Medicine Department, London, UK; 2 University College London, Great Ormond Street Institute for Child Health, London, UK; 3 Department of Development and Regeneration, Katholieke Universiteit Leuven, Leuven, Belgium; 4 Specialist Neonatal and Paediatric Surgery, Great Ormond Street Hospital NHS Trust, London, UK; 5 NIHR University College London Hospitals Biomedical Research Centre, Maple House, 149 Tottenham Court Road, London, W1T 7DN UK; 6 Institute for Stem Cell Research and Regenerative Medicine, Medical Faculty, Heinrich Heine University, Düsseldorf, Moorenstr. 5, 40225 Düsseldorf, Germany

Correction to: *Scientific Reports* 10.1038/s41598-019-44572-x, published online 31 May 2019

In the original version of this Article, the author Paolo De Coppi was incorrectly indexed. This error has now been corrected.

