# Supplementary material for: Human mid-trimester amniotic fluid (stem) cells lack expression of the pluripotency marker OCT4A
Source: Sci Rep. 2019 May 31;9:8126. doi: 10.1038/s41598-019-44572-x (PMC6544653; doi:10.1038/s41598-019-44572-x)
Supplement: Supplementary file 1 — Supplementary information [file 41598_2019_44572_MOESM1_ESM.pdf]

Human mid-trimester amniotic fluid (stem) cells lack expression of the pluripotency marker OCT4A.

Filipa Vlahova, Kate E Hawkins, Anna Maria Ranzoni, Kwan-Leong Hau, Rachel Sagar, Paolo De Coppi, Anna L David, James Adjaye and Pascale V Guillot

a

1 agagaggggt tgagtagtcc cttcgcaagc cctcatttca ccaggccccc ggcttggggc
61 gccttccttc cccatggcgg gacacctggc ttcggatttc gccttctcgc cccctccagg
121 tggtggaggt gatgggccag gggggccgga gccgggctgg gttgatcctc ggacctggct
exon 1 181 aagcttccaa ggccctcctg gagggccagg aatcgggccg gggttgggc caggctctga
241 ggtgtggggg attcccccat gcccccgcc gtatgagttc tgtgggggga tggcgtactg
301 tgggccccag gttggagtgg ggctagtgcc ccaaggcggc ttggagacct ctcagcctga
361 gggcgaagca ggagtcgggg tggagagcaa ctccgatggg gcctccccgg agccctgcac
421 cgtcacccct ggtgccgtga agctggagaa ggagaagctg gagcaaaacc cggaggagtc
exon 2 481 ccaggacatc aaagctctgc agaaagaact cgagcaattt gccaagctcc tgaagcagaa
541 gaggatcacc ctgggatata cacaggccga tgtggggctc accctggggg ttctatttgg
601 gaaggtattc agccaaacga ccatctgccg ctttgaggct ctgcagctta gcttcaagaa
exon 3 661 catgtgtaag ctgcggccct tgctgcagaa gtgggtggag gaagctgaca acaatgaaaa
721 tcttcaggag atatgcaaag cagaaaccct cgtgcaggcc cgaaagagaa agcgaaccag
781 tatcgagaac cgagtgagag gcaacctgga gaatttgttc ctgcagtgcc cgaaacccac
exon 4 841 actgcagcag atcagccaca tcgcccagca gcttgggctc gagaaggatg tggtccgagt
901 gtggttctgt aaccggcgcc agaagggcaa gcgatcaagc agcgactatg cacaacgaga
961 ggattttgag gctgctgggt ctcctttctc aggggggacca gtgtcctttc ctctggcccc
exon 5 1021 agggccccat tttggtaccc caggctatgg gagccctcac ttcactgcac tgtactcctc
1081 ggtccctttc cctgaggggg aagcctttcc ccctgtctcc gtcaccactc tgggctctcc
1141 catgcattca aactgaggtg cctgcccttc taggaatggg ggacaggggg aggggaggag
1201 ctagggaaag aaaacctgga gtttgtgcca gggtttttgg gattaagttc ttcattcact
1261 aaggaaggaa ttgggaacac aaagggtggg ggcaggggag tttggggcaa ctggttggag
1321 ggaaggtgaa gttcaatgat gctcttgatt ttaatcccac atcatgtatc actttttttct
1381 taaataaaga agcctgggac acagtagata gacacactta aaaaaaaaaa

b

Primer name	Primer sequence	Annealing temp	Amplicon size (bp)
OCT4A	F: 5’- CTT CTC GCC CCC TCC AGG T-3’<br>R: 5’- AAA TAG AAC CCC CAG GGT GAG C-3’	65	495
OCT4-Pg1	F: 5’-CATGCAGGCCCGAAAGAGAAAGCGAA- 3’<br>R: 5’- TGTGGCTGATCTGCAGTGTGGG- 3’	60	107
OCT4-Pg3	F: 5’- CTT CTC ACC CCC TCC AGG C- 3’<br>R: 5’- CCA CTG CTT GAT CGC TTG C- 3’	62	841
OCT4-Pg4	F: 5’-GGG ACA CCT GGC TTC GGA TG- 3’<br>R:CCC CAC ACC TCA GAG CCT GA-3’	56	172

Supplementary Figure 1: (a) Sequence of the OCT4A variant. **(b)** Sequence of primers used for qRT-PCR detection of the expression of OCT4A and pseudogenes 1 (OCT4A-Pg1), 3 (OCT4A-Pg3) and 4 (OCT4A-Pg4).

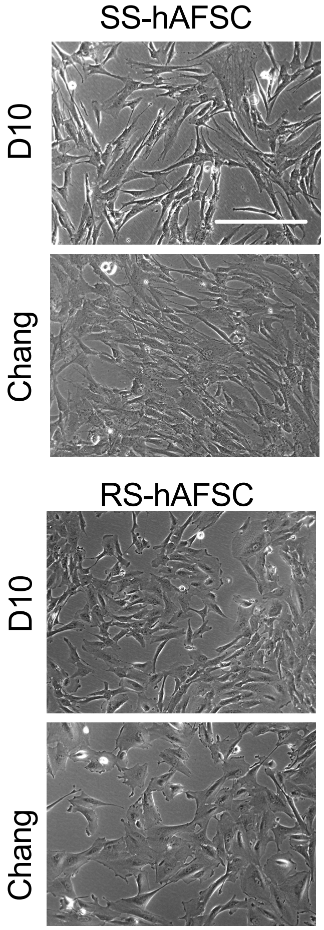

Supplementary Figure 2: Phase contrast images of RS-hAFSCs and SS-hAFSCs cultivated in Chang C or D10 culture medium. Scale bar = 100 μm

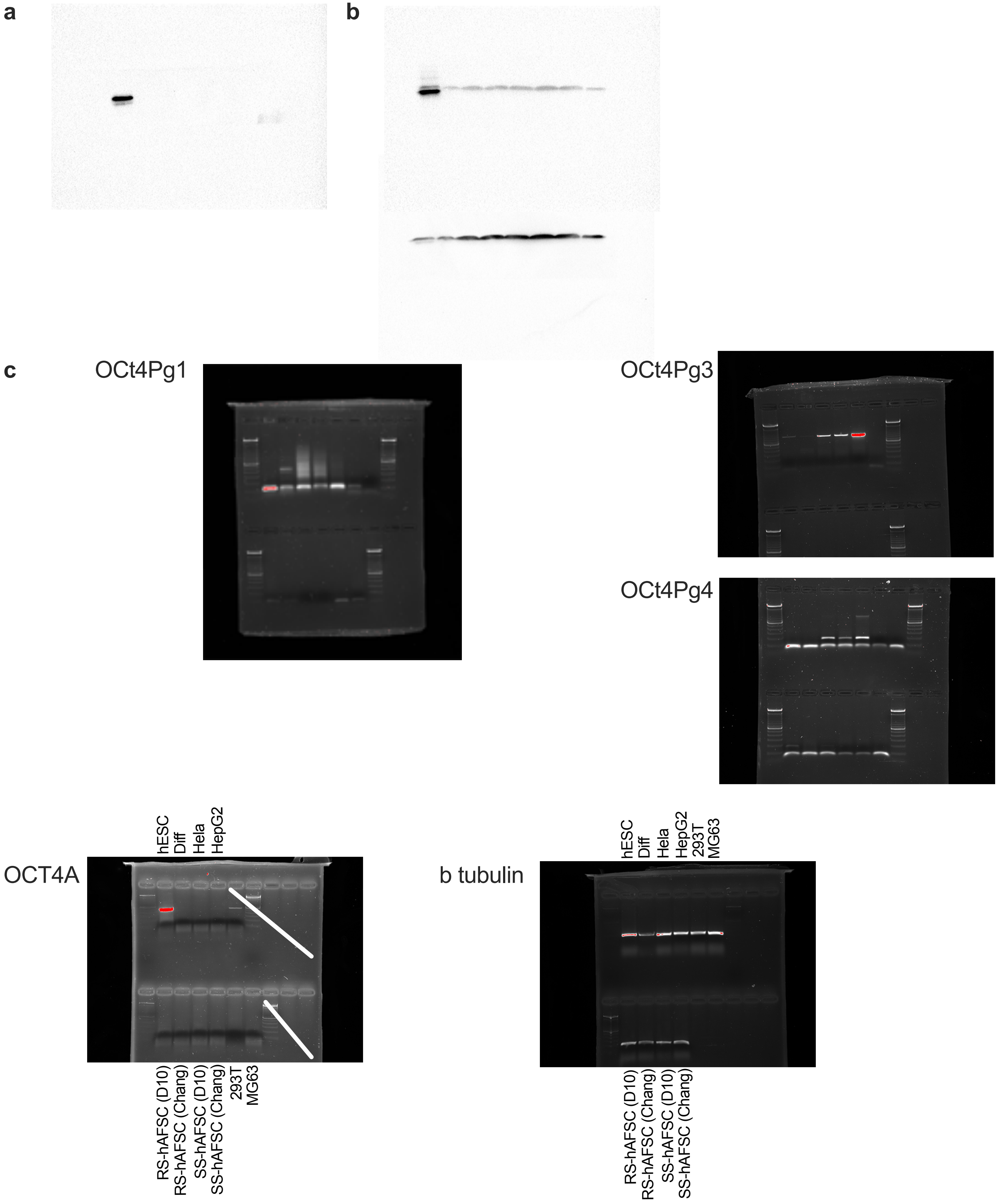

Supplementary Figure 3: Untouched blots corresponding to **(a)** Figure 3D left panel and **(b)** Figure 3d right panel, **(c)** Figure 6
